# Supplementary material for: The Use of Distinctive Monoclonal Antibodies in FMD VLP- and P1-Based Blocking ELISA for the Seromonitoring of Vaccinated Swine
Source: Int J Mol Sci. 2022 Aug 1;23(15):8542. doi: 10.3390/ijms23158542 (PMC9368795; doi:10.3390/ijms23158542)
Supplement: Supplementary file 1 [file ijms-23-08542-s001.zip › ijms-1841943-supplementary.pdf]

Supplementary Information

The Use of Distinctive Monoclonal Antibodies in FMD VLP- and P1-Based Blocking ELISA for the Seromonitoring of Vaccinated Swine

Heng-Wei Lee[1], Cheng-Yao Yang[2], Ming-Chang Lee[3], Shih-Ping Chen[3], Hui-Wen Chang[1,4], Ivan-Chen Cheng[1,*]

[1] School of Veterinary Medicine, National Taiwan University, Taipei 106, Taiwan

[2] Graduate Institute of Veterinary Pathobiology, National Chung Hsing University, Taichung City 402, Taiwan

[3] Agricultural Technology Research Institute, Hsinchu 300, Taiwan

[4] Graduate Institute of Molecular and Comparative Pathobiology, School of Veterinary Medicine, National Taiwan University, Taipei 106, Taiwan

[*]Corresponding author

Supplementary Figures S1–S3

Supplementary Tables S1–S2

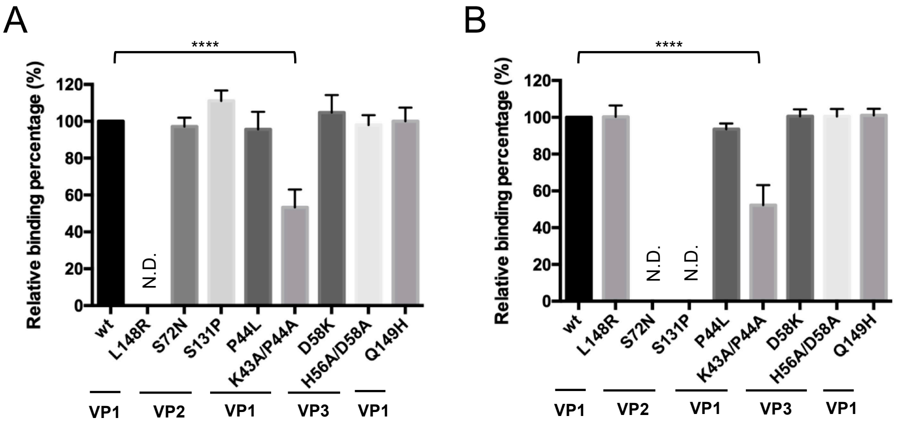

Figure S1. Relative binding percentages between S11B and each of the VLPs after normalization based on the OD values from (**A**) Q10E and (**B**) P11A. Site 1 mutated VLP was excluded from the normalization in Q10E because VP1-L148R would affect Q10E bindings. A similar situation was found for the normalization in P11A. ****: $p < 0.0001$ (One-way analysis followed by Dunnett’s multiple comparison test).

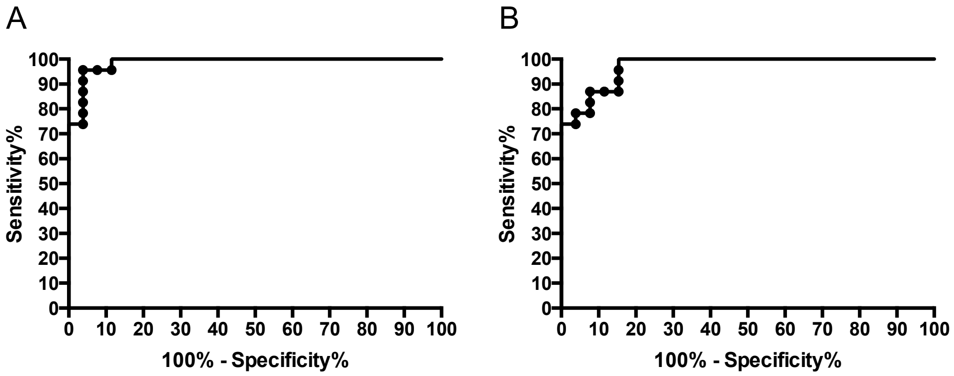

Figure S2. ROC analysis of (**A**) VLP:S11B-HRP and (**B**) P1:Q10E-HRP ELISA systems. The sensitivity and specificity were calculated using negative (n=26) and strong positive samples (n=23). The x-axis presents 100–specificity (%), and the y-axis shows sensitivity.

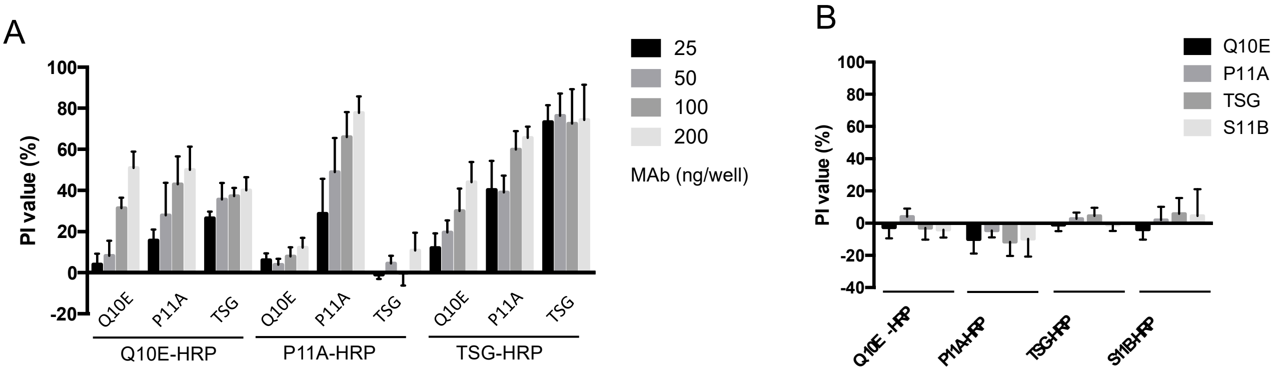

Figure S3. Steric effects between Q10E, P11A, and TSG on the P1 protein. (**A**) After being captured by anti-GST antibodies on the wells, GST-P1 were incubated with 25–200 ng of different non-conjugated antibodies, followed by tracers. (**B**) The change in the order of addition of non-conjugates and conjugates. After VLPs were captured, the indicated 100 ng of conjugates were added, followed by 400 ng of non-conjugates. PI values were calculated as regular blocking ELISA, and the values indicated the effect of replacement.

Table S1. Means of PI values for blocking ELISA in each pair.

			VLP				GST-P1		
Sample No.	SN	Log2 SN	Q10E-HRP	P11A-HRP	TSG-HRP	S11B-HRP	Q10E-HRP	P11A-HRP	TSG-HRP
SPF11	3	1.5849625	-2.0045906	1.81242872	1.047822	2.79499595	-0.5256136	0.94278426	1.2164628
SPF12	3	1.5849625	-3.0132273	2.95792544	2.15749226	3.71257375	3.39700143	3.18887047	2.79275545
SPF13	3	1.5849625	-2.3310841	3.85681809	3.14285106	7.13280387	8.27099187	1.97520762	2.05611558
SPF14	3	1.5849625	-1.4333943	5.22874885	3.73570247	4.02086502	9.89295684	4.52706496	2.29891973
SPF15	3	1.5849625	-0.3206017	6.09648406	1.96439507	5.17803109	2.65430311	3.43401288	0.20758324
SPF16	3	1.5849625	-1.6843161	2.18754738	2.7487305	2.6865123	1.9900009	0.8234575	1.64927617
SPF17	3	1.5849625	-3.4958884	2.23292271	1.45530678	0.98038126	10.0963463	5.55619323	8.58715147
SPF18	3	1.5849625	-0.851495	1.63023127	1.94433948	2.13658283	0.46525861	0.78831693	0.71407851
SPF19	3	1.5849625	-2.0233464	-0.3015908	0.05917242	1.50340096	1.19968337	1.39128516	-0.0999609
SPF20	3	1.5849625	-3.9312214	-1.5766654	-2.3186855	0.49143086	3.89009441	0.44300993	4.57247874
31	3	1.5849625	2.28056361	5.15270642	0.62657488	9.47112236	-0.7821576	2.56156523	2.30188192
33	3	1.5849625	4.89329202	7.411222	6.46944983	9.43172266	4.38085144	8.14077804	15.229887
36	3	1.5849625	-0.824548	2.81323191	2.69633957	3.94915731	5.34420608	5.8818745	11.7875165
38	3	1.5849625	12.2149113	12.7217724	27.26437	8.59305349	12.2629328	9.90817517	36.5595166
39	3	1.5849625	9.4511374	8.43131053	16.5273589	7.53771056	22.6809478	8.30728833	22.2635659
42	3	1.5849625	16.9890027	12.0264339	18.6216985	14.9284498	22.9899991	5.62822605	14.7438146
43	3	1.5849625	15.2795974	12.5067406	26.9424882	8.78286065	23.9738524	10.6032821	30.0488402
44	4	2	19.2277644	15.0913792	24.8562445	22.1093467	27.2131388	7.89423404	26.619596
50	4	2	18.0264019	17.8849053	44.409872	13.1293904	40.0601072	21.2330225	71.6619341
40	8	3	38.6159753	26.9818422	62.8545643	28.7568601	40.1732842	25.3416489	74.6431143
47	8	3	21.3233607	19.9838587	31.4434859	16.9143303	30.6274867	17.8585973	47.6572526
48	8	3	33.3131058	20.1878664	48.5994364	29.5855154	47.3481159	23.6322179	68.6339656
54	8	3	44.1022626	40.3341072	46.4186156	45.8527282	50.0367511	43.5437211	68.726349
82	8	3	10.8811035	8.92864991	14.1458932	21.5412433	6.15210273	5.6109807	8.65710646
85	8	3	14.3812596	5.82407543	15.502514	12.2065061	11.2794837	3.42387286	22.0209817
86	8	3	16.0786688	8.67211407	23.5572499	9.35581614	12.2752518	6.04604464	23.3549333
45	16	4	21.2117231	18.4098665	31.3958446	24.4841608	29.3895264	15.4530207	43.7545712
46	16	4	23.6852626	14.3550343	20.1836735	29.1845855	22.5306814	5.63354952	18.9860087
49	16	4	28.2634281	20.1607031	33.5132046	21.8953324	40.1678868	25.7112897	56.4913605
58	16	4	13.7589845	12.9059065	24.3484373	13.1365224	32.9115928	14.7340697	38.6537858
59	16	4	34.1484647	30.8875403	40.4103577	49.5810663	44.660209	26.9241703	63.8056237
60	16	4	16.2663045	13.1320907	50.7435716	16.7538363	40.0797829	22.3334084	71.6363639
88	16	4	17.9300904	16.8629663	19.7802387	18.8961474	23.1205114	22.9461562	51.1926199
51	32	5	30.3019245	27.7078745	37.2501803	31.3515344	52.659482	30.6914553	62.0751955
55	32	5	33.153533	23.5969293	49.6865569	58.5713006	31.7826557	23.2098185	67.7698849
57	32	5	32.44168	25.5480902	43.8839019	39.273757	37.1957415	22.6174101	69.4077111

72	32	5	25.4788903	26.0429312	30.9983537	29.4913454	35.1067288	22.9114416	39.6063358
81	32	5	25.0297898	15.6464786	27.8668894	26.7543884	32.6437077	14.6156662	33.4261192
84	32	5	42.663149	23.1487669	38.6442401	47.2050417	36.5652751	20.7247809	54.3551524
87	32	5	11.2488749	3.1413975	10.8475549	6.84075041	25.9849304	12.0727588	30.6209029
52	64	6	25.2872125	22.506115	28.3829183	36.5344885	39.097605	20.4724954	58.0993923
76	64	6	35.80888	26.0029583	48.451231	37.9302707	47.5479035	38.2604764	75.8365411
80	64	6	47.2423102	47.9649424	55.1612947	47.4356779	54.3637038	47.1221374	80.191068
83	64	6	40.4774722	24.1369934	41.3754367	42.6046535	36.1328519	19.7496446	51.8878015
89	64	6	27.7677125	35.4316139	65.3051079	45.3350381	54.4748809	39.1422732	74.0302139
53	128	7	28.1941771	24.3428583	33.0248604	38.8029642	41.007982	19.2660635	59.926556
56	128	7	20.3449109	11.6884276	23.6910334	28.6091294	36.6053864	14.8727882	39.9453016
62	128	7	44.8129017	37.3893543	61.4539406	62.9371297	46.9541314	29.3085364	70.5691195
64	128	7	69.8390376	50.3074538	78.0847657	72.0814298	59.3664122	37.6029278	77.6297409
69	128	7	36.1514948	17.8569041	54.3559027	56.8725832	60.2007176	18.5978734	65.3459958
70	128	7	40.6381129	33.1386817	50.7020235	52.6618259	51.9183131	23.9168156	59.5868472
71	128	7	60.9089806	34.0785477	61.8414413	73.4334345	52.2251876	24.2361843	73.3559294
73	128	7	47.3871916	35.2065494	57.8545772	75.0725335	56.288024	26.9184481	72.6325299
77	128	7	49.4840519	46.0470479	58.6364615	57.3429467	61.1240674	38.9720816	81.3424764
78	128	7	58.2006267	52.8711886	68.7304139	77.0559754	57.7455199	37.5547433	81.2434429
41	256	8	83.3325765	71.232493	84.5476906	87.9368206	78.439414	62.3878033	88.8818456
65	256	8	52.2326308	69.0872352	59.1425513	54.6369914	65.5907828	65.3494923	90.2784991
66	256	8	77.9645925	68.3326557	87.2620967	82.9644019	77.578644	53.7980943	90.0567548
79	256	8	78.5869048	66.9149368	63.8204509	75.3616763	71.8951302	44.8658145	68.2408993
61	512	9	48.7458969	42.8216786	41.1218242	59.4868463	54.7230737	24.1831045	70.2678939
63	512	9	76.0741351	60.0358542	77.4455664	77.6725827	75.6588662	48.4882273	81.3206522
67	512	9	77.0887861	81.0026857	83.1642554	83.0055658	72.0564904	67.5995457	85.0504492
68	512	9	64.5001866	46.6082356	66.5151942	69.2939135	57.6804622	38.7292395	75.6216825

The values for false positive samples were labeled in blue, while false negative represented in red.

Table S2. Mutagenic primers.

	Name	Sequence (5'-3')
**Site 1**	VP1-L148R-F	ACGTGAGGGGTGACCGTCAAGTGTTAGCTCA
	VP1-L148R-R	TGAGCTAACACTTGACGGTCACCCCTCACGT
**Site 2**	VP2-S131P-F	ACCGGAGCTCTGTCCCATCAGCAAGAGAGA
	VP2-S131P-R	TCTCTCTTGCTGATGGGACAGAGCTCCGGT
**Site 3**	VP1-P44L-F	TCGTGAAAGTCAAGCTAAAGGAACAAGTTAA
	VP1-P44L-R	TTAACTTGTTCCTTTAGCTTGACTTTCACGA
	VP1-K43A/P44A-F	CAGGTTCGTGAAAGTCGCCGCAAAGGAACAAGTTA
	VP1-K43A/P44A-R	TAACTTGTTCCTTTGCGGCGACTTTCACGAACCTG
**Site 4**	VP3-D58K-F	GCCCCACATTCCTACACTTCAAAGGTGACGTTC
	VP3-D58K-R	GAACGTCACCTTTGAAGTGTAGGAATGTGGGGC
	VP3-H56A/D58A-F	TGCCCCACATTCCTAGCCTTCGCCGGTGACGTTCCGTA
	VP3-H56A/D58A-R	TACGGAACGTCACCGGCGAAGGCTAGGAATGTGGGGCA
**Site 5**	VP1-Q149H-F	GAGGGGTGACCTTCACGTGTTAGCTCAGAAG
	VP1-Q149H-R	CTTCTGAGCTAACACGTGAAGGTCACCCCTC

The nucleotide that has been changed was shown in red.
